# Interspecific variation and phylogenetic relationship between mangrove and non-mangrove species of a same family (Meliaceae)—insights from comparative analysis of complete chloroplast genome

**DOI:** 10.7717/peerj.15527

**Published:** 2023-06-26

**Authors:** Fengxiao Tan, Weixi Li, Hui Feng, Yelin Huang, Achyut Kumar Banerjee

**Affiliations:** 1 College of Natural Resources and Environment, South China Agricultural University, Guangzhou, Guangdong, China; 2 School of Life Sciences, Sun Yat-Sen University, Guangzhou, Guangdong, China

**Keywords:** Phylogenetics, Mangroves, Coastal ecosystem, Chloroplast genome, Population genetics, Adaptation, Stress response

## Abstract

The mahogany family, Meliaceae, contains 58 genera with only one mangrove genus: *Xylocarpus*. Two of the three species of the genus *Xylocarpus* are true mangroves (*X. granatum* and *X. moluccensis*), and one is a non-mangrove (*X. rumphii*). In order to resolve the phylogenetic relationship between the mangrove and non-mangrove species, we sequenced chloroplast genomes of these *Xylocarpus* species along with two non-mangrove species of the Meliaceae family (*Carapa guianensis* and *Swietenia macrophylla*) and compared the genome features and variations across the five species.

The five Meliaceae species shared 130 genes (85 protein-coding genes, 37 tRNA, and eight rRNA) with identical direction and order, with a few variations in genes and intergenic spacers. The repetitive sequences identified in *the rpl22* gene region only occurred in *Xylocarpus*, while the repetitive sequences in *accD* were found in *X. moluccensis* and *X. rumphii*. The *TrnH-GUG* and *rpl32* gene regions and four non-coding gene regions showed high variabilities between *X. granatum* and the two non-mangrove species (*S. macrophylla* and* C. guianensis*). In addition, among the *Xylocarpus* species, only two genes (*accD* and *clpP*) showed positive selection. *Carapa guianensis* and *S. macrophylla* owned unique RNA editing sites. The above genes played an important role in acclimation to different stress factors like heat, low temperature, high UV light, and high salinity. Phylogenetic analysis with 22 species in the order Sapindales supported previous studies, which revealed that the non-mangrove species *X. rumphii* is closer to *X. moluccensis* than *X. granatum*. Overall, our results provided important insights into the variation of genetic structure and adaptation mechanism at interspecific (three *Xylocarpus* species) and intergeneric (mangrove and non-mangrove genera) levels.

## Introduction

Mangrove forests consist of an important group of woody plants occupying coastal zone habitats (Tomlinson, 2016). They are vital intertidal wetland ecosystems and directly impact the welfare of coastal communities in the tropics and subtropics (Ge & Sun, 1999). Their occupation of the tidal zone is manifested in a range of specialized attributes, such as viviparous embryos, tolerance for salt fluctuations and uncommon root systems (Tomlinson, 2016). Understanding their evolutionary history, molecular basis of the adaptation mechanisms across the coastal zone habitats, and genetic divergence from the non-mangrove species are some of the central themes of mangrove species research. The chloroplast DNA (cpDNA hereafter) is heavily used for evolutionary studies and phylogenetic analysis (*e.g.*, Asaf et al., 2021). The cpDNA offers a highly conserved sequence due to uniparental inheritance, and haploid and non-recombinant nature (Daniell et al., 2016). Due to these properties, it has now become a powerful tool in accurate reconstruction of phylogenetic relationships (Li et al., 2019; Muellner et al., 2003).

In this study, we focused on the genus *Xylocarpus*, which has three species with accepted names (the name currently accepted as the one which should be used in preference to refer to a species): *X. granatum* J.Koenig, *X. moluccensis* (Lam.) M.Roem., and *X. rumphii* (Kostel.) Mabb. (https://powo.science.kew.org/taxon/urn:lsid:ipni.org:names:26860-1#children). Among these three species, *X. granatum* (also known as ‘cedar mangrove’, ‘cannonball tree’ or ‘puzzle nut tree’) is the most widespread species in East Africa, tropical Australia, Southeast Asia, and the Pacific Islands (Waratchareeyakul et al., 2017). This evergreen species is usually distributed in mangrove fringe or the nearby sandy shores, and can grow up to 15 m in height (Tomlinson, 2016). The second species, *X. moluccensis* (also known as ‘apple mangrove’) is distributed from the East-Africa to Philippines, Australia and the Pacific Islands (Haron, 2010). This small tree of 3–10 m height usually grows on sandy shores. The third species, *X. rumphii*, is a rare species of 4–15 m in height and its distribution is restricted to tropical Asia, Africa and Australia (Waratchareeyakul et al., 2017). This species does not grow in mangrove ecosystem, rather occurs above high-water level on cliffs, rocks, and sandy upland areas (Waratchareeyakul et al., 2017). Among the three species, *X. granatum* and *X. moluccensis* are considered as true mangrove species based on their occurrence only in mangrove environment (*i.e.,* in tidal swamps), and possession of specialized morphological features (like aerial roots and vivipary of the embryo) and physiological mechanism for salt exclusion and/or excretion (Tomlinson, 2016).

The genus *Xylocarpus* is one of the 58 genera of the mahogany family, Meliaceae, and is the only genus that contains two mangrove species (Muellner-Riehl & Rojas-Andrés, 2022). Previous molecular marker assisted analysis based on three plastid DNA sequences (*trnL-F*, *rbcL*, and *atpB*) revealed that four non-mangrove species of the family Meliaceae, namely *Carapa guianensis*, *Swietenia macrophylla*, *Swietenia mahagoni*, and *Khaya nasica*, formed a cluster in a clade with *Xylocarpus* (Muellner-Riehl et al., 2016). However, the study only considered one *Xylocarpus* species (*X. moluccensis*). The phylogenetic pattern became different when all three *Xylocarpus* species and five other cpDNA loci (*psbA-trnH*, *trnC-ycf6*, *trnH-trnK*, *trnS-trnG* and *trnT-trnL*) were considered in another study (Guo et al., 2018). Overall, the phylogenetic relationship between the mangrove and non-mangrove species of Meliaceae remains inconsistent.

In this context, analysis of complete chloroplast genomes can provide important insights into the evolutionary relationships between phylogenetic clades, as observed for several plant genera (*e.g.*, Huang et al., 2014) and families (*e.g.*, Lin et al., 2018). In addition, comparative analysis of chloroplast genomes can reveal variation in genome sequences and structures, both within and between plant species. This information can be valuable for advanced understanding of the adaptive ability of species in face of different environmental conditions and biotic and abiotic stresses, which may facilitate conservation of valuable traits (Daniell et al., 2016). Given that complete chloroplast genome sequence can improve our understanding of plant evolution and have several translational applications, it is not surprising that about 4,000 chloroplast genomes have been sequenced to date (Singh et al., 2020).

This study was therefore formulated to characterize the complete chloroplast genomes of three *Xylocarpus* species (including two true mangroves and one non-mangrove species) and two non-mangrove species from the same family. We did not use nuclear markers (like 26S, or ITS), as these markers were found to be not sufficiently informative (like 26S) and contain saturated substitutions at family level (like ITS) for Sapindales (Muellner-Riehl et al., 2016). We selected *C. guianensis* and *S. macrophylla* as the two non-mangrove species, as they were found to be phylogenetically closest to the *Xylocarpus* species in the previous studies (Guo et al., 2018; Muellner-Riehl et al., 2016). We further compared the sequence genome features and variations between the true mangrove and non-mangrove species to provide the molecular insights into the adaptive mechanisms of the mangrove species. Although complete chloroplast genome information is available for *C. guianensis* in GenBank (NC_037442) and for *X. moluccensis* in CNSA (CNP0000567) (Shi et al., 2020), we characterized them in this study to avoid influence of different methodologies on the comparative analysis.

## Materials & Methods

### DNA extraction and sequencing

Young leaves of three *Xylocarpus* species, *C. guianensis* and *S. macrophylla* were collected from Singapore (sample collection was approved by the National Parks Board, Singapore, with the permit No: NP/RP14-023) and China, respectively, after confirming their taxonomic identities (Tomlinson, 2016). The voucher specimens were deposited in the herbarium of Sun Yat-sen University, and the collection information for these samples are listed in Table S1. The leaves were stored at −80 °C. Total DNA were extracted by CTAB protocol (Doyle & Doyle, 1990). The pair-end (PE) libraries with 150bp insert size were prepared in Vazyme Co. Ltd (Nanjing, China) and their sequences were conducted on the Illumina HiSeq X Ten platform. In total, 9.5G, 12.2G, 12.5G, 10.6G, 11.7G shorts sequences of *X. granatum*, *X. moluccensis*, *X. rumphii, C. guianensis* and *S. macrophylla* were respectively obtained.

### Chloroplast genome assembling and annotation

Raw reads of the five Meliaceae species were filtered to remove the low-quality bases, and the clean reads were assembled to chloroplast genome in NOVOPlasty (Dierckxsens, Mardulyn & Smits, 2017). The chloroplast genomes of five species were annotated in the DOGMA program (Wyman, Jansen & Boore, 2004) using the whole chloroplast genome of *C. guianensis* (GenBank accession number: NC_037442.1) as a reference. Some unannotated genes were further manually adjusted by Geneious R version 10.1.3 (Biomatters Ltd., Auckland, New Zealand). To validate the accuracy of assembling, we designed four pairs of primers in four junctions between the large single-copy region (LSC hereafter), single small copy region (SSC hereafter), and two inverted repeats (IRA and IRB hereafter) (Table S2). PCR amplification was conducted followed by Sanger sequencing. We further verified the start-and-stop codons of the coding sequences (CDS hereafter) and exon boundaries through BLASTX in NCBI GenBank/DDBJ/EMBL database, and confirmed tRNA through online tRNAscan-SE web servers (Schattner, Brooks & Lowe, 2005). Finally, a circle chloroplast genome map was drawn by the online program OGDraw version 1.1 (Lohse, Drechsel & Bock, 2007). The annotated chloroplast genome sequences of all five species were deposited to GenBank with the following accession numbers: MH348115 (*X. granatum*), MH330688 (*X. moluccensis*), MH330687 (*X. rumphii*), MH396436 (*C. guianensis*), and MH348156 (*S. macrophylla*).

### Identification of repeat sequences and simple sequence repeats

The repeat sequences in the chloroplast genomes of the five study species were identified in the online program REPuter (Kurtz et al., 2001). Palindromic, forward, and reverse sequences were identified with the setting of maximum repeat size 3 kb and minimum repeat size 30 bp, based on a Hamming distance of 3. To avoid redundancy, IRB regions of the genomes were removed. Overlapping repeats discovered in the same region were merged into one repeat when possible. SSRs are tracts of repetitive DNA with motif length from two to five base pairs, and are commonly used as genetic markers to assess genetic diversity and distinguish varieties within species (Ellegren, 2004). Identification of SSRs was performed by using the WebSat software (Martins et al., 2009) with the following settings: three repeats for hexa-nucleotide, three repeats for penta-nucleotide, three repeats for quad-, four repeats for tri-, five repeats for di-, and 10 repeats for mono-nucleotide SSRs.

### Genome-wide sequence variations and gene selective pressure analysis

We used the Snippy program (https://github.com/tseemann/snippy) to analyze the SNPs and INDEL among five study species. The divergence among the species was visualized using the mVISTA viewer and the genome-wide sequence variation was computed using *X. granatum* as a reference. Variation percentages in coding regions and non-coding regions were calculated. By using the PAMLX version 1.3.1 (Xu & Yang, 2013), the relative evolution rate of the five chloroplast genomes was quantified based on dN, dS and their ratios (*ω* = dN/dS) (Yang & Nielsen, 2000) with the F 3 × 4 codon-based substitution model. After deleting the stop codons, 85 protein-coding genes were aligned using MEGA version 6.06 (Tamura et al., 2013). For each protein-coding genes, we estimated dN, dS and *ω* and compared between the five species.

### Codon usage and RNA editing sites

The codon usage of 85 protein coding genes was identified by using codonW version 1.4.2 with RSCU ratio under default settings (http://codonw.sourceforge.net). We selected coding genes of at least 300 bp to avoid the codon bias index associated with short sequences. In addition, the repeat sequences were removed to avoid repetitive computation. A total of 85 CDS of every species were used in PREP suit (https://bio.tools/prep_suite) to predict possible RNA edit sites in PREP-cp (for chloroplast genes). The cutoff value was selected as 0.8 to ensure the accuracy of the prediction (Mower, 2009).

### Phylogenetic analysis

Genome sequence of the five study species were aligned along with 18 other species using MAFFT version 7.310 (Katoh & Standley, 2013) with default settings, and then trimmed using trimAl version 1.4 (Capella-Gutiérrez, Silla-Martínez & Gabaldón, 2009). Out of the 18 species, 16 species were selected from the order Sapindales, whereas two species were selected from the related orders Brassicales and Malvales and were treated as outgroups following (Muellner-Riehl et al., 2016) (Table S2). The maximum likelihood phylogenetic tree was inferred in IQ-TREE version 2.0.3 (Nguyen et al., 2015) under TVM+F+I+G4 model with 1000 bootstraps. Bayesian inferences (BI) were conducted by the MPI version of ExaBayes version 1.5.1 (Aberer, Kobert & Stamatakis, 2014) under an automatically created model by the Program. By sampling every 1000 generations, four independent MCMC runs with two chains were carried out from parsimony starting topologies. Until the mean topological differences was met with at least 1,000,000 generations, the ExaBayes runs were continued. The ‘consense’ script with a 25% burn-in was used to summarize the posterior distributions of the trees. When the effective sampling sizes (ESS) of all the parameters were greater than 200, convergence was assumed. The convergence was tested with Tracer version 1.7 (Rambaut et al., 2018). The potential scale reduction factors (PSRF) close to 1 was estimated using the ‘postProcParam’ program in ExaBayes. Both ML and BI tree were visualized by using Figtree version 1.4.2 (Rambaut, 2014).

## Results

### Characteristics of the chloroplast genomes

The complete cpDNA length of the five Meliaceae species ranged from 159,276 bp of *S. macrophylla* of to 159,467 bp of *C. guianensis* (Table S3). All of them showed a typical quadripartite structure, containing a pair of inverted repeats (IRs: IRA and IRB) (26,967–27,052 bp), which were separated by one large single copy region (LSC) (86,958–87,381 bp) and one small single copy region (SSC) (17,967–18,276 bp). The cpDNA length of three *Xylocarpus* species were different: 159,282 bp of *X. rumphii*, 159,319 of *X moluccensis* and 159,410 of *X. granatum*. We found 429, 412 and 159 SNPs or INDEL between *X. granatum vs. X moluccensis*, *X. granatum vs. X. rumphii* and *X. rumphii vs. X moluccensis*, respectively (Table S4). The earlier accession of *C. guianensis*, collected from French Guiana, was sequenced by Malte Msder (2018, NCBI Accession number: NC_037442). In this study, we collected sample from Sungei Buloh Wetland Reserve, Singapore, which was planted from Cooperative Republic of Guyana on 28 December 1938. We found 12 SNPs or INDEL between the earlier accession and our sample (Table S4), which indicated that the two sequences were nearly identical.

**Figure 1:**
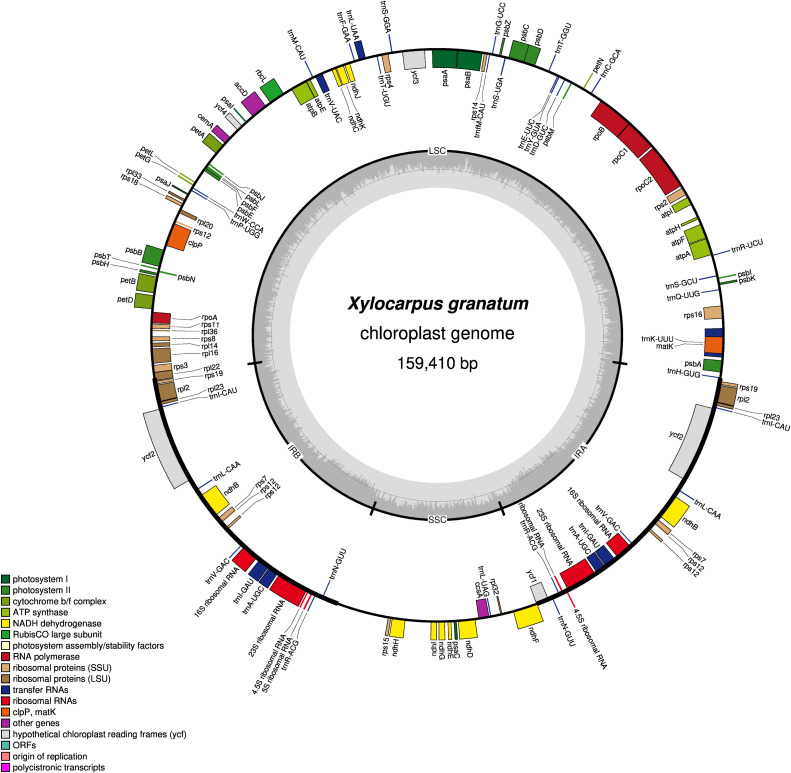
Gene map of chloroplast genomes of *Xylocarpus granatum*. Genes showing outside of the outer layer circle are transcribed clockwise direction, whereas those inside genes are transcribed counterclockwise. Different functional groups are represented by the colored bars. The darker gray area lying in the inner circle indicates GC content of the complete chloroplast genome.

The GC contents of the three species were nearly identical (GC contents of *X. moluccensis* and *X. rumphii* were 37.87%, GC content of *X. granatum* was 37.86%). The other two species of the genus Meliaceae, namely *S. macrophylla* and *C. guianensis*, had 37.95% and 37.91% GC content, respectively. A total of 130 functional genes were found in each of the study species: 85 protein-coding genes (CDS), 37 tRNA and 8 rRNA (Table 1). Out of these, 81 genes were in LSC region, and 13 genes were found in SSC region. A total of 18 genes (seven protein-coding genes, seven tRNA and four rRNA) was duplicated in the IR regions (Fig. 1, Figs. S1–S4). Among the 130 genes, 15 genes included one intron and three genes (*rps12*, *ycf3* and *clpP*) included two introns. The *rps12* was a trans-spliced gene and had two directions with 5′ exon in LSC region and 3′ end exon in IR region. In addition, *rps19* and *ndhD* genes contained unusual start codons—GTG and ACG, respectively. 58 genes (four rRNA, 25 protein-coding genes and 29 tRNA) were related to self-replication (Table 1). Out of the 46 genes associated with photosynthesis, 22 genes were related to the subunits of photosystem, 11 genes to NADH dehydrogenase, six genes to the subunits of cytochrome, six genes to the subunits of ATP synthase, and only one gene was related to the large subunit of rubisco. The rest of the genes encoded proteins (*e.g.*, *clpP* encoding Protease, *matK* encoding Maturase, *cemA* encoding envelop membrane protein). The functions of some genes could not be deciphered (*accD*, *ccsA*, *ycf1* and *ycf2*) (Table 1).

**Table 1:** List of genes present in three *Xylocarpus* species and two relatives: *Carapa guianensis* and *Swietenia macrophylla*.

**Category**	**Group**	**genes**
Self-replication	Ribosomal RNA genes	*rrn4.5* (×2)*, rrn5* (×2)*, rrn16* (×2)*, rrn23* (×2)
	Large subunit of ribosome (LSU)	*rpl2* (×2)^*^, rpl14, rpl16^*^, rpl20, rpl22, rpl23 (×2)*, rpl32, rpl33, rpl36*
	small subunit of ribosome (SSU)	*rps2, rps3, rps4, rps7* (×2), rps8, rps11, rps12^**^, rps14, rps15, rps16^*^, rps18, rps19 (×2)
	DNA dependent RNA polymerase	rpoA, rpoB, rpoC1^*^, rpoC2
	Transfer RNAs genes	*trnA-UGC(*×2)^*^, trnC-GCA, trnD-GUC, trnE-UUC, trnF-GAA, trnG-UCC(×2)^*^, trnH-GUG, trnI-CAU(×2*), trnI-GAU(*×2)^*^, trnK-UUU^*^, trnL-CAA(×2), trnL-UAA^*^, trnL-UAG, trnM-CAU, trnfM-CAU, trnN-GUU(×2*), trnP-UGG, trnQ-UUG, trnR-ACG(*×2*), trnR-UCU, trnS-GCU, trnS-UGA, trnS-GGA, trnT-UGU, trnT-GGU, trnV-GAC(*×2), trnV-UAC^*^, trnW-CCA, trnY-GUA
Photosynthesis	Subunits of photosystem I	psaA, psaB, psaC, psaI, psaJ, ycf3^**^, ycf4
	Subunits of photosystem II	*psbA, psbB, psbC, psbD, psbE, psbF, psbH, psbI, psbJ, psbK, psbL, psbM, psbN, psbZ, psbT*
	NADH dehydrogenase	ndhA^*^, ndhB(×2)^*^, ndhC, ndhD, ndhE, ndhF, ndhG, ndhH, ndhI, ndhJ, ndhK
	Subunits of cytochrome	petA, petB^*^, petD^*^, petG, petL, petN
	Subunits of ATP synthase	atpA, atpB, atpE, atpF^*^, atpH, atpI,
	Large subunit of rubisco	*rbcL*
Other genes	ATP-dependent protease subunit p gene	clpP^**^
	Maturase	*matK*
	Envelop membrane protein	*cemA*
Unknown function	Subunit of acetyl-CoA-carboxylase	*accD*
	c-type cytochrome synthesis gene	*ccsA*
	Hypothetical chloroplast reading frames	*ycf1* (×2)*, ycf2* (×2)

**Notes.**

* Gene with one intron.

** Gene with two introns, (×2) Gene with two copies.

### Repetitive sequences

**Figure 2:**
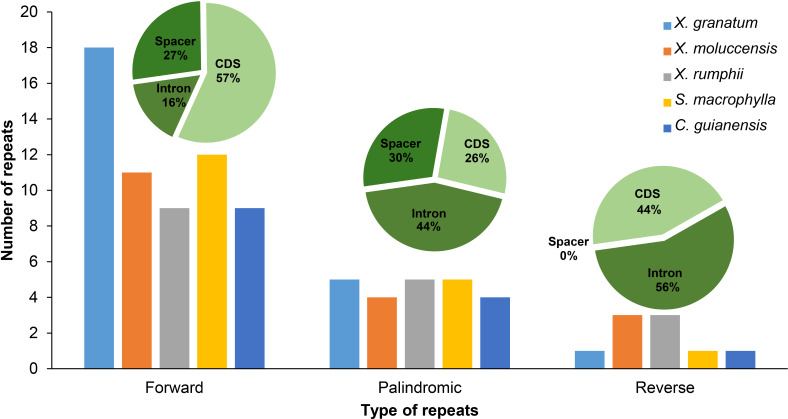
Number of three types of repeats (forward, palindromic and reverse) in five Meliaceae species in three different locations. protein coding genes (cds), gene intron (intron) and intergenic spacer (spacer). Five species: *Xylocarpus granatum*, *Xylocarpus moluccensis*, *Xylocarpus rumphii*, *Swietenia macrophylla*, *Carapa guianensi*.

Three types of repetitive sequences were identified in the five Meliaceae species. *X. granatum* possessed highest number of repetitive sequences (*n* = 24) among the five species studied here. *X. moluccensis* and *X. rumphii* contained equal number of reverse repeats (*n* = 3); however, quantities of forward and reverse types were different between the two species. We found 11 forward repeats and four palindromic repeats in *X. moluccensis*, and nine forward repeats and five palindromic repeats in *X. rumphii*. The two related species of this family, *S. macrophylla* and *C. guianensis*, had almost identical repeats distribution. Comparing all repeat type sequences, the figure for palindromic appeared to be almost stable among these five species. However, forward repeats ranged from 10 in *C. guianensis* to 18 in *X. granatum*, and reverse repeats ranged from 1 (*X. granatum, S. macrophylla*, *C. guianensis*) to 3 (*X. moluccensis and X. rumphii*)*.* The distributions of three type of repeats were different in each region: approximately half of forward repeats located in the CDS region, while 27% and 16% of the repeats were distributed in the spacer region and intron region, respectively. In palindromic repeats, 44% repeats were situated in the intron region, while 30% and 26% repeats were identified in the spacer region and CDS region, respectively. No repeats were discovered in spacer region of reverse repeats, while 56% and 44% repeats were found in intron region and CDS region, respectively (Fig. 2, Table S5).

In *X. granatum*, nine repeats (64% of total CDS) were located in the *rpl22* gene region, while in *X. moluccensis* and *X. rumphii*, the number of repeats in the *rpl22* gene region were 2 and 1, respectively. No repeat was found in the *rpl22* gene region for *S. macrophylla* and *C. guianensis*. At *ycf2* gene, one repeat was founded for the three *Xylocarpus* species, while four and three repeats were observed in *S. macrophylla* and *C. guianensis*, respectively. We found variable number of repeats (three to four) in the *ycf3* intron range among these five Meliaceae species. At *accD* gene, two reverse repeats were found in *X. moluccensis and X. rumphii*, and these repeats were not identified in other species. Majority of these repeats were found in the CDS of the genome. Maximum number of repeats was observed in *X. granatum* (14), followed by *X. moluccensis* (nine), *X. rumphii* (seven), *S. macrophylla* (seven) and *C. guianensis* (five). Palindromic repeats were discovered in CDS, gene intron (intron) and intergenic spacer (spacer) regions, while reverse repeats were only found at CDS and intron regions (Fig. 2, Table S5).

### SSR analysis

**Figure 3:**
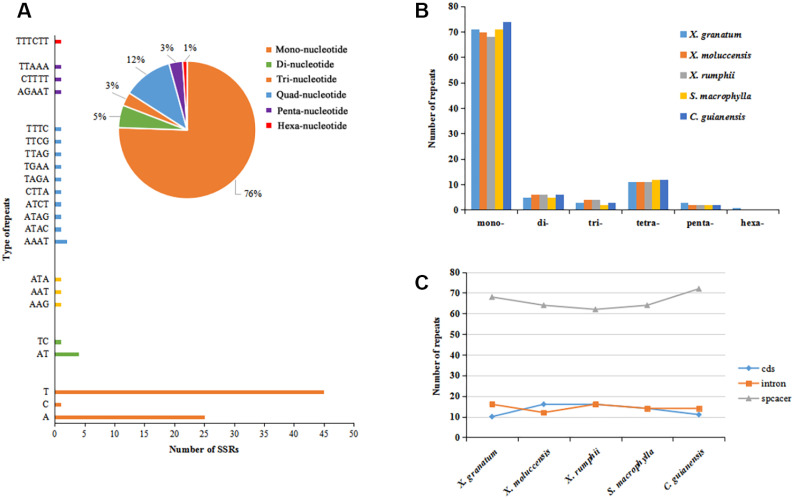
Analysis of simple sequence repeat (SSR) type. (A) Distribution of different SSRs repeat units in *Xylocarpus granatum*, (B) number of repeats for six types of SSRs in five Meliaceae species (*Xylocarpus granatum*, *Xylocarpus moluccensis*, *Xylocarpus rumphii*, *Swietenia macrophylla*, *Carapa guianensis*), (C) variation in number of repeats among five Meliaceae species in three different locations: protein coding genes (cds), gene intron (intron) and intergenic spacer (spacer).

A total of 467 simple sequence repeats (SSRs, also known as microsatellites) were identified across the five study species. The number of SSRs varied from 92 in *X. moluccensis* and *S. macrophylla* to 97 in *C. guianensis* (Table S6). In all the five species, the number of mononucleotide SSRs was maximum (ranging from 68 to 74, accounting for 74% to 77% in all repeats) (Figs. 3A–3B, Fig. S5). Other types of SSRs constituted a small part with 113 repeats comprising of 11-12 tetra-nucleotide SSRs, 5-6 di-nucleotide SSRs, 2-3 penta-nucleotide SSRs, and 1 hexa-nucleotide SSRs (only found in *X. granatum*). Most of these SSRs were located in the intergenic spacer region. We found 68 SSRs (72.3%), 64 SSRs (69.6%), 62 SSRs (66.0%), 64 SSRs (69.6%), 72 SSRs (74.2%) in the intergenic spacer regions of *X. granatum*, *X. moluccensis*, *X. rumphii*, *S. macrophylla* and *C. guianensis*, respectively (Fig. 3C). It is interesting to note that almost identical number of SSRs was observed in coding sequences (10-16) and intron sequences (12-16).

### IR contraction and expansion

**Figure 4:**
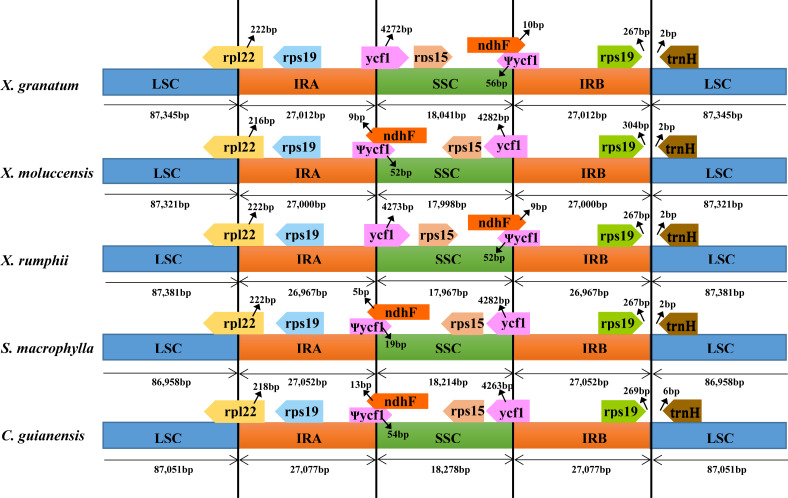
Comparison of large single-copy (LSC), small single-copy (SSC), and inverted repeat (IR) border regions among the chloroplast genomes of five Meliaceae species and one closely related species. Five Meliaceae species: *Xylocarpus granatum*, *Xylocarpus moluccensis*, *Xylocarpus rumphii*, *Swietenia macrophylla*, *Carapa guianensis*, one closely related species: *Ailanthus altissima* in Simaroubaceae.

To identify contraction and expansion of the IR region, the IR boundaries were compared between these five Meliaceae species and one closely related species *Ailanthus altissima* of the family Simaroubaceae (GenBank accession number: MG799542). We observed highly conserved IR boundary regions with a little variation in length of the boundary genes (Fig. 4). For all the six Sapindales species, the LSC region started with *trnH* gene and ended in 3′ region of *rpl22*. The *trnH* gene of three *Xylocarpus* species and *S. macrophylla* started at position 3, whereas in *C. guianensis* and *A. altissima*, it started at position 7 and 21, respectively. IRA region started from 5′ region of *rpl22* with 222 bp in *X. granatum*, *X. rumphii* and *S. macrophylla*, and with 216 bp in *X. moluccensis,* 218 bp in *C. guianensis* and 6bp in *A. altissima*. Two copies of *rps19* were located in the start region of IRA and in the reverse region of IRB. The IRA/SSC junctions were situated within an overlap region between pseudogene *ycf1* and *ndhF* in all five study species*.* The overlap regions ranged from 24bp in *S. macrophylla* to 67bp in *X. granatum* and *C. guianensis*. When compared with *A. altissima*, *ndhF* gene was not found to be located in the IRA/SSC junction and shared no nucleotide with *ycf1* pseudogene. The *ycf1* genes were situated in the 3′ region in SSC of the SSC/IRB border with 4263–4328 bp length, followed by the *rps15* gene which was completely located in the SSC regions.

### Genome-wide sequence variations and positive selection test

**Figure 5:**
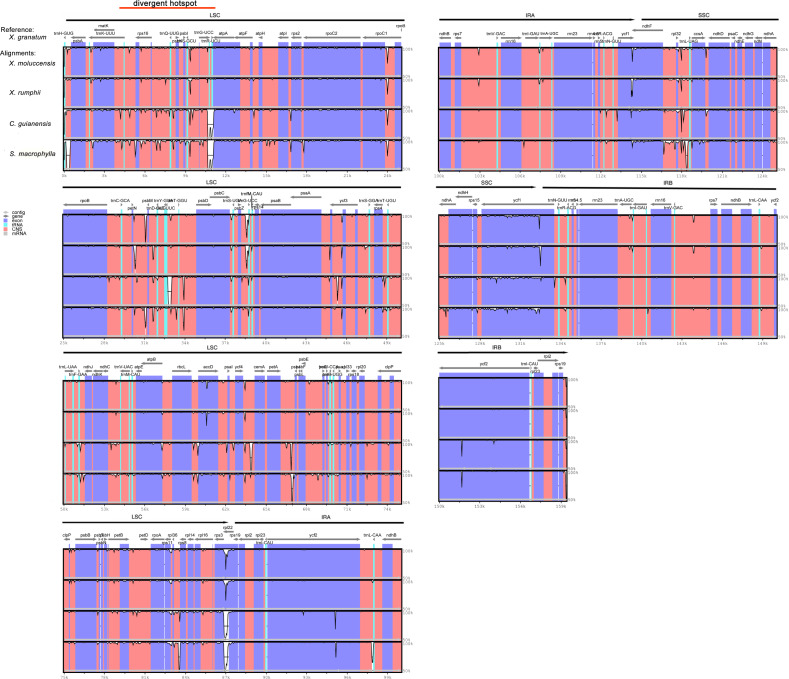
Visualization of complete chloroplast genome alignments of five Meliaceae species using *Xylocarpus granatum* as a reference. VISTA-based identity plots showing the identity between five species on *y*-axis (between 50%–100%). *X*-axis showed the consensus sequences location of chloroplast genomes. Different colors represented different regions: protein coding genes (exon): purple, conserved noncoding sequences (CNS): red, tRNA coding regions (tRNA): blue.

**Figure 6:**
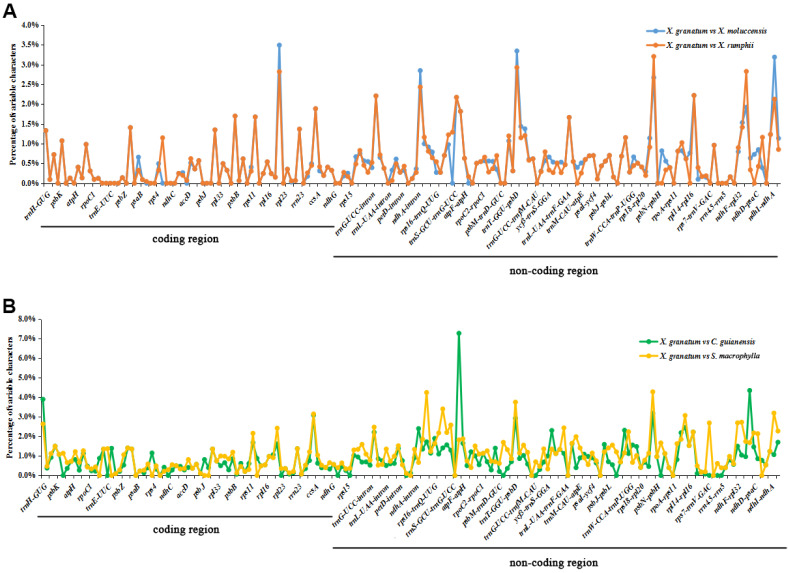
Percentages of variable characters in aligned regions between five Meliaceae species chloroplast genomes using *Xylocarpus granatum* as a reference. (A) Variation between *X. granatum* and two mangrove species (*Xylocarpus moluccensis* and *Xylocarpus rumphii*), (B) variation between* X. granatum* and two non-mangrove species (*Carapa guianensis* and *Swietenia macrophylla*).

The analysis of genome divergence level between the five study species revealed that the sequences were highly conserved with only a few divergences in some regions (Fig. 5). The differences were mainly distributed in the LSC and SSC regions. One divergent hotspot in the LSC region (*trnK-UUU* - *atpA*) was shown with a red line in Fig. 5. Non-coding regions exhibited higher variability compared to that observed in the coding regions. Only three genes showed >3% variability in the coding regions with variability ranging from 3.90% between *X. granatum* and *C. guianensis* in *trnH-GUG*, 3.49% between *X. granatum* and *X. moluccensis* in *rpl22*, 3.09% between *X. granatum* and *C. guianensis*, and 3.14% between *X. granatum* and *S. macrophylla* in *rpl32*. On the other hand, four non-coding regions showed more than 4% variability: 4.24% between *X. granatum* and *S. macrophylla* in *trnK-UUU*—*rps16*, 7.27% between *X. granatum* and *C. guianensis* in *atpA* - *atpF*, 4.28% between *X. granatum* and *S. macrophylla* in *psbB* - *psbT*, and 4.35% between *X. granatum* and *C. guianensis* in *ccsA-ndhD* (Fig. 6, Table S7).

Analysis of the relative evolution rate revealed that only two genes (*accD* and *clpP*) showed positive selection between the three *Xylocarpus* species (Table S8). *X. granatum vs. X. moluccensis* showed *ω* = 1.1667 and *ω* = 7.0625 in *accD* and *clpP*, respectively. *X. granatum vs. X. rumphii* showed similar result with *ω* = 1.3333 and *ω* = 7.0625 in *accD* and *clpP*, respectively. Notably, all pairwise genes of *X. moluccensis vs. X. rumphii* showed *ω*<1.

### Codon usage frequency and putative RNA editing sites analysis

**Figure 7:**
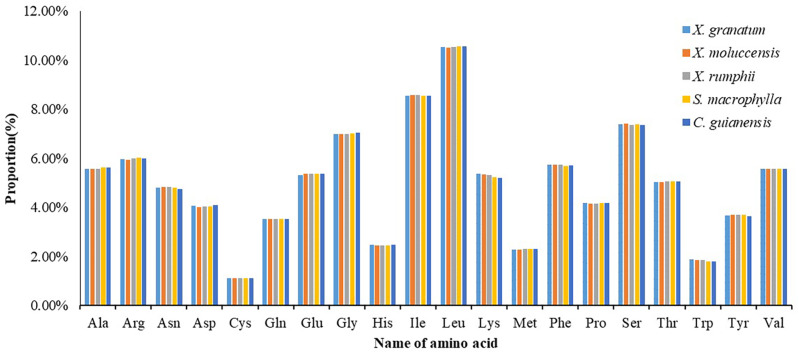
Numbers of genes under positive selection (*ω* > 1) among pairwise comparisons of five Meliaceae specie. *Xylocarpus granatum*, *Xylocarpus moluccensis*, *Xylocarpus rumphii*, *Swietenia macrophylla*, *Carapa guianensis*.

All 85 protein coding genes were extracted and then combined into consecutive protein-coding genes for each of the five species (lengths varied from 21,554 to 21,706) (Table S9). Analysis of the codon usage frequency and relative synonymous codon usage of the five species revealed that all species allocated a similar share of the amino acids (Fig. 7, Table S9). The most abundant amino acid was Leucine (Leu) (10.5%), followed by Isoleucine (Ile) (8.5%) and Serine (Ser) (7.4%). The amino acid Cystine (Cys) constituted the minimum proportion (1.1%) (Fig. 7). For almost all A/U-ending amino acids, relative synonymous codon usage (RSCU) values exceeded 1, while the CUA (encode Leu) and AUA (encode Ile) values were less than 1 (Table S9). Conversely, RSCU values for most of the G/C-ending amino acids were always less than 1. Two amino acids, namely Threonine (Trp) and Methionine (Met), showed no codon bias.

The comparative estimates of RNA prediction editing sites of the five study species were found to be similar to each other. All three species in genus *Xylocarpus* showed an identity prediction, implying that they were highly conserved (Table S9). Compared to the *Xylocarpus* species, the *ndhF* and *rpoC2* genes lacked the GCC =>GTC sites in *C. guianensis*. Besides, *S. macrophylla* had two unique RNA editing events: one in *ccsA* gene: GCC =>GTC, and another in *ndhF* gene: CTT =>TTT. Except these disparities, all five study species were predicted with same RNA editing sites and scores. A total of 53 RNA editing sites in 20 genes were shared by the five chloroplast genomes (Table 2). Out of these 53 RNA editing sites, 40 occurred at the second position of these codons, while only 13 occurred at the first positions. No RNA editing site was found at the third position of these codons. It is notable that two genes, namely *ndhB* and *ndhD*, had 10 editing sites each (maximum number among these protein codon genes) (Table S10).

**Table 2:** Predicted RNA editing sites shared list of the Five Meliaceae species chloroplast genome protein coding genes by PREP program.

**Gene**	**Align collective position**	**Effect**	**Score**
*accD*	290	T CG (S) =>T TG (L)	0.8
*atpA*	264	C CC (P) =>C TC (L)	1
*atpB*	135	CCA (P) =>TCA (S)	0.86
*atpF*	31	C CA (P) =>C TA (L)	0.86
*clpP*	187	CAT (H) =>TAT (Y)	1
*matK*	165	CAC (H) =>TAC (Y)	1
	196	CAT (H) =>TAT (Y)	0.86
	227	CAT (H) =>TAT (Y)	1
*ndhA*	36	C CT (P) =>C TT (L)	1
	114	T CA (S) =>T TA (L)	1
	189	T CA (S) =>T TA (L)	1
	358	T CC (S) =>T TC (F)	1
*ndhB*	50	T CA (S) =>T TA (L)	1
	156	C CA (P) =>C TA (L)	1
	196	CAT (H) =>TAT (Y)	1
	204	T CA (S) =>T TA (L)	0.8
	246	C CA (P) =>C TA (L)	1
	249	T CT (S) =>T TT (F)	1
	277	T CA (S) =>T TA (L)	1
	279	T CA (S) =>T TA (L)	1
	419	CAT (H) =>TAT (Y)	1
	494	C CA (P) =>C TA (L)	1
*ndhD*	10	A CG (T) =>A TG (M)	1
	114	CGG (R) =>TGG (W)	0.8
	137	C CA (P) =>C TA (L)	1
	234	T CA (S) =>T TA (L)	1
	302	T CA (S) =>T TA (L)	1
	305	C CT (P) =>C TT (L)	1
	368	G CT (A) =>G TT (V)	1
	442	T CA (S) =>T TA (L)	0.8
	446	T CA (S) =>T TA (L)	0.8
	478	CTT (L) =>TTT (F)	0.8
*ndhF*	347	A CT (T) =>A TT (I)	1
	648	G CT (A) =>G TT (V)	0.8
*ndhG*	56	CAT (H) =>TAT (Y)	0.8
	107	A CA (T) =>A TA (I)	0.8
*psbF*	26	T CT (S) =>T TT (F)	1
*rpl20*	103	T CA (S) =>T TA (L)	0.86
*rpoA*	279	T CA (S) =>T TA (L)	1
*rpoB*	113	T CT (S) =>T TT (F)	1
	185	T CA (S) =>T TA (L)	1
	190	T CG (S) =>T TG (L)	1
	662	G CT (A) =>G TT (V)	0.86
	827	T CA (S) =>T TA (L)	0.86
*rpoC1*	21	T CA (S) =>T TA (L)	1
*rpoC2*	584	CAT (H) =>TAT (Y)	0.86
	862	A CT (T) =>A TT (I)	1
	959	CGG (R) =>TGG (W)	1
	1288	CTT (L) =>TTT (F)	0.86
*rps14*	27	T CA (S) =>T TA (L)	1
	53	T CA (S) =>T TA (L)	1
*rps16*	71	T CA (S) =>T TA (L)	0.83
*rps2*	83	T CA (S) =>T TA (L)	1

### Phylogenetic analysis

**Figure 8:**
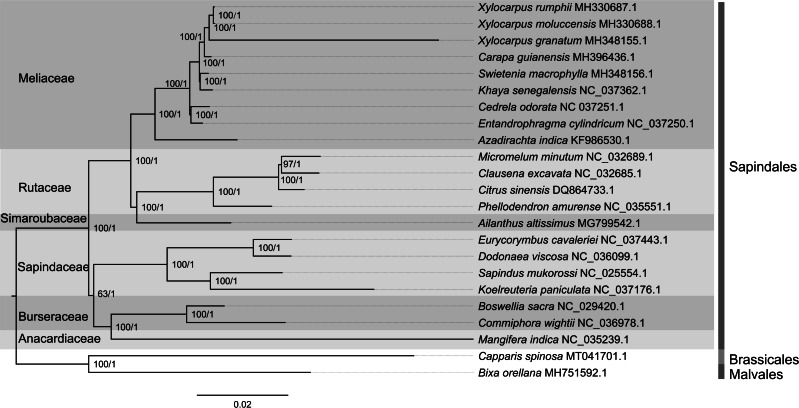
Phylogenetic relationship of 21 species in the order Sapindales and two outgroup species based on whole chloroplast genomes. Support values are showed at each node: the former is maximum likelihood bootstrap and the latter is Bayesian inference posterior probability.

Identical phylogenetic positions of the five study species were obtained from maximum likelihood (ML) and Bayesian inference (BI) methods. Each of the 22 species from six families of the order Sapindales clustered within their own families and each clade was highly supported by both phylogenetic analysis methods. Majority of the nodes in the ML tree were well supported with bootstrap value higher than 95%, except the most recent common ancestor (MRCA) of Burseraceae, namely Anacardiaceae and Sapindaceae. In BI tree, each node was supported with posterior probability of 1. In both phylogenetic trees, three *Xylocarpus* species were clustered together, *X. moluccensis* and *X. rumphii* clustered as one clade, while *X. granatum* was sister to this clade (Fig. 8). The *Xylocarpus* species clade formed cluster with *C. guianensis*. *Swietenia macrophylla* was found to be the sister branch of *Khaya senegalensis* with 100% bootstrap support and clustered with the *Xylocarpus* and *Carapa* clade. The topologies in genus level were consistent with the previous research (Lin et al., 2018). In addition, the Meliaceae clade was sister to the clade of two families, namely Simaroubaceae and Rutaceae.

## Discussion

This is the first study which reported the whole chloroplast genome sequences of three *Xylocarpus* species, including the rare non-mangrove species *X. rumphii* and two true mangrove species. In addition, the study also reported the genome sequences of two closely related non-mangrove species of *Xylocarpus*, among which *S. macrophylla* is considered as a vulnerable species (https://www.iucnredlist.org/species/32293/9688025; accessed on 12 April 2023). Our study, therefore, adds novel information to the whole genome sequences of the order Sapindales and family Meliaceae.

Analysis of the interspecific variation in chloroplast genome sequences revealed that the five Meliaceae species showed similarity in GC contents and gene features. All genes shared identical direction and order, with only a few variations in intergenic spacer sequences. The chloroplast genomes in Meliaceae were highly conserved and no obvious contraction or expansion were found in IR borders.

### Intraspecific variation based on repeat sequences

While the GC contents and gene features showed the conservatism of chloroplast genomes in Meliaceae, the distribution pattern of repeat sequences explained the divergence of *Xylocarpus* from the other two species and between the three *Xylocarpus* species themselves. Our study identified presence of three repetitive sequences across five Meliaceae species. Based on the similar proportion of repeats among the three species of *Xylocarpus*, high similarity of chloroplast genomes among these species became evident.

However, *X. granatum* was found to have the highest number of repeats in *rpl22*, while only two repeats and one repeat were found in *X. moluccensis* and *X. rumphii*, respectively. No repeats were observed in *C. guianensis* and *S. macrophylla*. It has been reported that *rpl22* gene was more likely to be transferred to nuclear genome from chloroplast genome (Gantt et al., 1991), which could increase genomic variation and diversity and consequently, enhance the adaptability of a species to adverse environmental conditions. Comparing the niche width (*i.e.,* a metric to ascertain the adaptability of species to tolerate environmental conditions) between 29 species from the mangrove ecosystem of the Bhitarkanika sanctuary (Odisha, India), Upadhyay & Mishra (2014) showed that *X. granatum* had larger niche width (2.15) than *X. moluccensis* (1.15). The existence of more repetitive sequences in *rpl* 22, therefore, might explain the wide distribution of *X. granatum*, compared to the other two *Xylocarpus* species, and might also account for the divergence between *X. granatum* and other four Meliaceae species (two *Xylocarpus* species and two non-mangrove species) studied here.

Finally, we observed similar repeat sequences in *acc* D in *X. moluccensis* and *X. rumphii*. The gene *acc* D is associated with leaf form and photosynthetic efficiency, since presence of *acc* D in plastids promotes gene expression in light-harvesting organs (Kode et al., 2005). Similar repeat sequences in this gene in *X. moluccensis* and *X. rumphii* may cause similar leaf appearances in these two species (Li, Su & Wang, 2018). Overall, our findings provided strong support to the previous studies which indicated that repeat sequences may play an important role in rearranging and recombination of chloroplast genomes (Asano et al., 2004; Zeng et al., 2017). The phylogenetic analysis also revealed clustering of the five study species into three clades (one *Xylocarpus* clade and other two relative clades) with the relationship between *X. moluccensis* and *X. rumphii* being closer than *X. granatum*.

In addition to providing insights into the divergence pattern of the five species of Meliaceae, the repeat sequences, especially the simple sequence repeats (SSRs), provided powerful genetic resources for investigating population diversity. In this study, we identified 467 SSR loci, most of which showed polymorphisms among the five study species. These loci, therefore, could act as molecular markers for investigating population genetics and biogeography in Meliaceae family. The advantage of using cpDNA SSR marker is that it allows for the detection of population genetic structure and gene flow at the maternal level, which may be informative for understanding the demographic history and evolutionary relationships of these species. Moreover, in terms of feasibility, the use of cpDNA SSR markers, or cpDNA markers as a whole, is relatively straightforward and cost-effective, as it requires amplification and sequencing of a single locus. However, using only one marker may not provide a complete picture of the population genetics of a species or group, as different markers may capture different aspects of genetic diversity and evolutionary history.

### Intraspecific variation based on coding and non-coding regions

The analysis of genome divergence revealed >3% variability in the coding regions and >4% variability in four non-coding regions between *X. granatum* and other four Meliaceae species (two *Xylocarpus* species and two non-mangrove species) studied here. Two out of three coding genes, namely *rpl22* and *rpl32*, are associated with the large subunit of ribosome. Previous studies have identified that ribosomal proteins of the large subunit 22 and 23 are essential under heterotrophic conditions (Fleischmann et al., 2011). This genomic divergence pattern may explain the enhanced adaptability of *X. granatum* to a wide range of environmental conditions. In addition, these gene spacer regions can be used as genetic markers in future phylogenetic and molecular taxonomy studies of mangrove species.

A number of genes under positive selection were also identified in this study which highlighted the evolutionary aspects of these species. Among these genes, notable was the *clpP* gene, which showed a positive *ω* value for all three *Xylocarpus* species. Earlier studies reported that ATP-dependent *Clp* protease is essential for acclimation to different stress factors like temperature, radiation and salinity (Ostersetzer & Adam, 1996; Porankiewicz, Schelin & Clarke, 1998; Porankiewicz, Wang & Clarke, 1999; Zheng et al., 2002). A previous study based on near-chromosome-scale genome analysis also revealed signals of positive selection in genes related to salt tolerance and root development in the three species of *Xylocarpus* (Guo et al., 2022). Our finding, therefore, might explain the wide adaptability of the *Xylocarpus* species to tropics and subtropical coastal zone habitats, which are continuously subjected to physiological stresses caused by fluctuating environmental conditions (*e.g.*, inundation, sudden alteration of salinity and temperature levels) (Fusi et al., 2022).

In coding sequences of the five study species, the usage frequencies of the most A/U-ending amino acids were higher than that of the G/C-ending amino acids. In addition, all the putative editing sites were C-to-U transitions, and majority of these sites were found to be situated in the second positions of these codons. These findings provide evidence of certain biasness in codon editing of chloroplast genomes. Our codon usage frequency results are consistent with previous studies, which showed the similar amino acids distribution among coding sequences of Sapindales members (*e.g.*, Saina et al., 2018).

Most editing sites variation may convert the original amino acids to hydrophobic amino acids, which may be conducive to the formation of protein stable structure and provided a certain basis for the plants in adapting to the environment. The number of editing sites of *ndhB* and *ndhD* genes was found to be maximum in all five Meliaceae species, which might be because they participate in multiple biological processes, and their expression and regulatory activity were stronger than others. In coding sequences of the five Meliaceae species, the most abundant amino acid was Leu, a plant growth promoter having photosynthetic and regulating effect. In addition, differences in editing sites were found between the three *Xylocarpus* species and the two non-mangrove species (*C. guianensis* and *S. macrophylla*), which might contribute to the divergence between the mangrove and non-mangrove species of Meliaceae.

### Phylogenetic analysis

The two phylogenetic analysis methods (ML and BI) revealed identical topological structure. Interestingly, the phylogenetic tree showed closer relationship of the mangrove species *X. moluccensis* to the non-mangrove species *X. rumphii*, rather than another mangrove species *X. granatum*. This finding is in line with the previous phylogenetic pattern based on specific cpDNA and nDNA loci sequences (Guo et al., 2018). One possible explanation could be drawn from the recently proposed pre-adaptation framework based on near-chromosome-scale genome analysis, which also revealed similar phylogenetic pattern for *Xylocarpus* (Guo et al., 2022). According to this framework, the common ancestor of *Xylocarpus* lived close to the coasts; *X. granatum* and *X. moluccensis* were subsequently adapted to the intertidal habitat due to the mangrove-specific adaptive features like preferential retention of duplicated genes and intolerance of pseudogenization, while *X. rumphii* retained the pre-adaptive features and were adapted to the terrestrial habitat (Guo et al., 2022). However, chloroplast genomes are more conserved than the nuclear genome and therefore, frequency of changes in chloroplast genomes are lower compared to the nuclear genomic changes. Therefore, we observed positive selection in only two genes, namely *accD* and *clpP*, showed positive selection between the three *Xylocarpus* species, and no genes were found under positive selection in *X. moluccensis vs. X. rumphii*.

## Conclusions

To the best of our knowledge, this is the first study that characterized complete chloroplast genomes for five Meliaceae species and identified the intraspecific variations between genomes of two mangrove and three non-mangrove species. Based on the distribution pattern of repetitive sequences, variability in coding and non-coding regions, differences in editing sites in coding sequences and phylogenetic analysis, our study identified the divergence pattern between the three *Xylocarpus* species (two mangroves and one non-mangrove species) and two related non-mangrove species of Meliaceae and supported the previous observations. Our study also highlighted the importance of repetitive sequences and genes under positive selection in stress response and adaptation of the three *Xylocarpus* species, especially that of the *X. granatum*, in fluctuating environmental conditions. Finally, the polymorphic simple sequence repeats and gene regions identified in this study can act as genetic markers in future molecular taxonomy research for other members of the Meliaceae family.

##  Supplemental Information

10.7717/peerj.15527/supp-1 Supplemental Information 1 Species information used in this study

10.7717/peerj.15527/supp-2 Supplemental Information 2 List of PCR primer sets for assembly validation

10.7717/peerj.15527/supp-3 Supplemental Information 3 The basic chloroplast genome characteristics of five Meliaceae species

10.7717/peerj.15527/supp-4 Supplemental Information 4 SNP and INDEL among three Xylocarpus species and two accessions of Carapa guianensis

10.7717/peerj.15527/supp-5 Supplemental Information 5 Repeats (¿= 30bp) identified in five Meliaceae species

10.7717/peerj.15527/supp-6 Supplemental Information 6 SSR position in five Meliaceae species chloroplast genome

10.7717/peerj.15527/supp-7 Supplemental Information 7 Percentage of variation sites in each region

10.7717/peerj.15527/supp-8 Supplemental Information 8 Nonsynonymous substitution (dN), Synonymous substitution (dS), and dN/dS (*ω* ) values for individual genes or gene groups

10.7717/peerj.15527/supp-9 Supplemental Information 9 Codon usage in all protein coding genes of five Meliaceae species

10.7717/peerj.15527/supp-10 Supplemental Information 10 Putative RNA editing sites

10.7717/peerj.15527/supp-11 Supplemental Information 11 Gene map of chloroplast genomes of *Xylocarpus moluccensis*

10.7717/peerj.15527/supp-12 Supplemental Information 12 Gene map of chloroplast genomes of *Xylocarpus rumphii*

10.7717/peerj.15527/supp-13 Supplemental Information 13 Gene map of chloroplast genomes of *Carapa gulanensis*

10.7717/peerj.15527/supp-14 Supplemental Information 14 Gene map of chloroplast genomes of *Swietenia macrophylla*

10.7717/peerj.15527/supp-15 Supplemental Information 15 Distribution of different SSRs repeat units of 4 species other than *Xylocarpus granatum*: a) *Xylocarpus moluccensis*, b) *Xylocarpus rumphii*, c) *Swietenia macrophylla* and d)* Carapa gulanensis*

## Additional Information and Declarations

### Field Study Permissions

The following information was supplied relating to field study approvals (i.e., approving body and any reference numbers): Field experiments were approved by the National Parks Board, Singapore (NP/RP14-023).

### DNA Deposition

The following information was supplied regarding the deposition of DNA sequences: The annotated genome sequences of all five species are available in GenBank: MH348115 (*X. granatum*), MH330688 (*X. moluccensis*), MH330687 (*X. rumphii*), MH396436 (*C. guianensis*), and MH348156 (*Swietenia macrophylla*).

### Data Availability

The following information was supplied regarding data availability: The data are available in the Supplemental Files.
