# Supplementary figures and images for: Interspecific variation and phylogenetic relationship between mangrove and non-mangrove species of a same family (Meliaceae)—insights from comparative analysis of complete chloroplast genome
Source: PeerJ. 2023 Jun 26;11:e15527. doi: 10.7717/peerj.15527 (PMC10309054; doi:10.7717/peerj.15527)

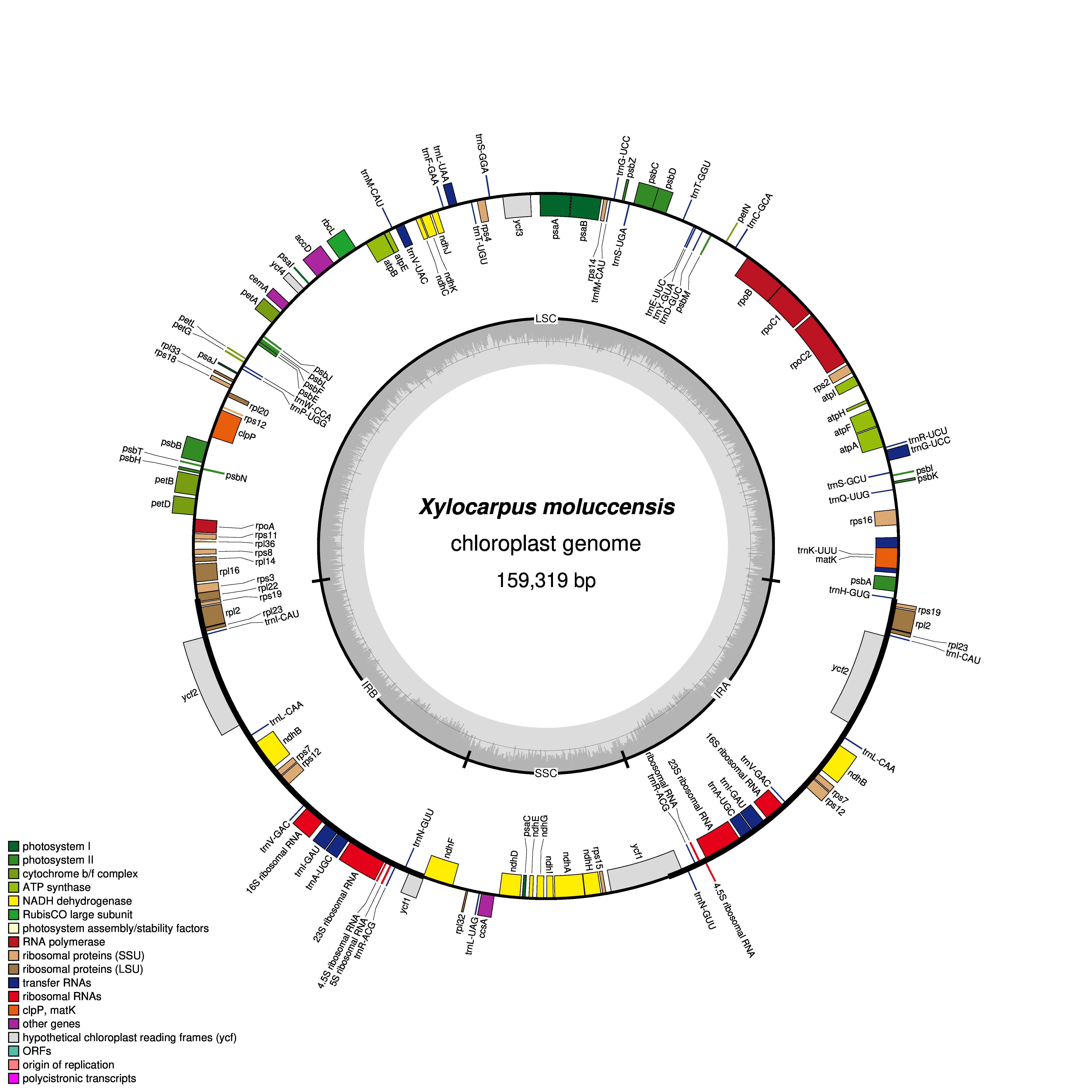

Supplement: Supplemental Information 11 [file peerj-11-15527-s011.jpg]

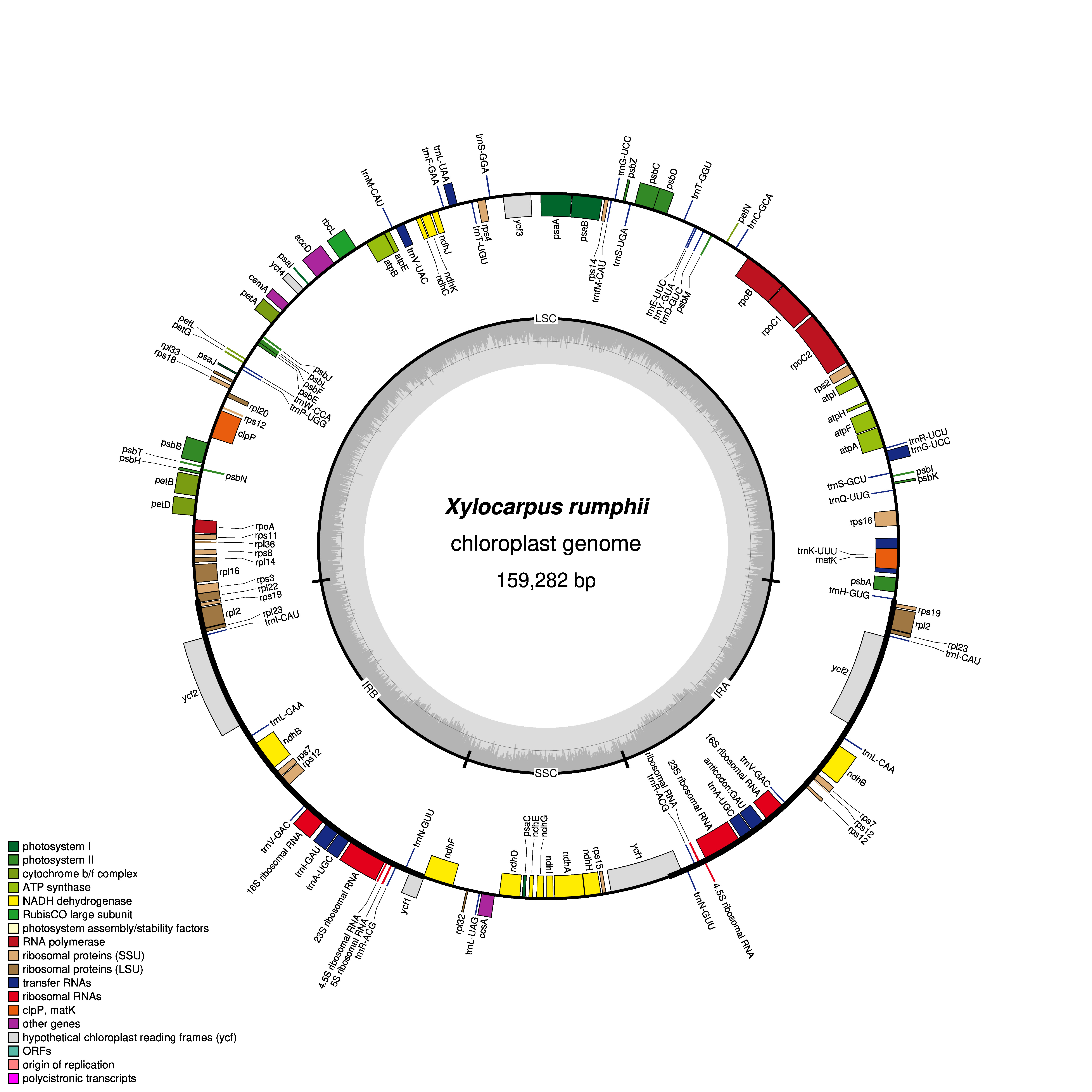

Supplement: Supplemental Information 12 [file peerj-11-15527-s012.jpg]

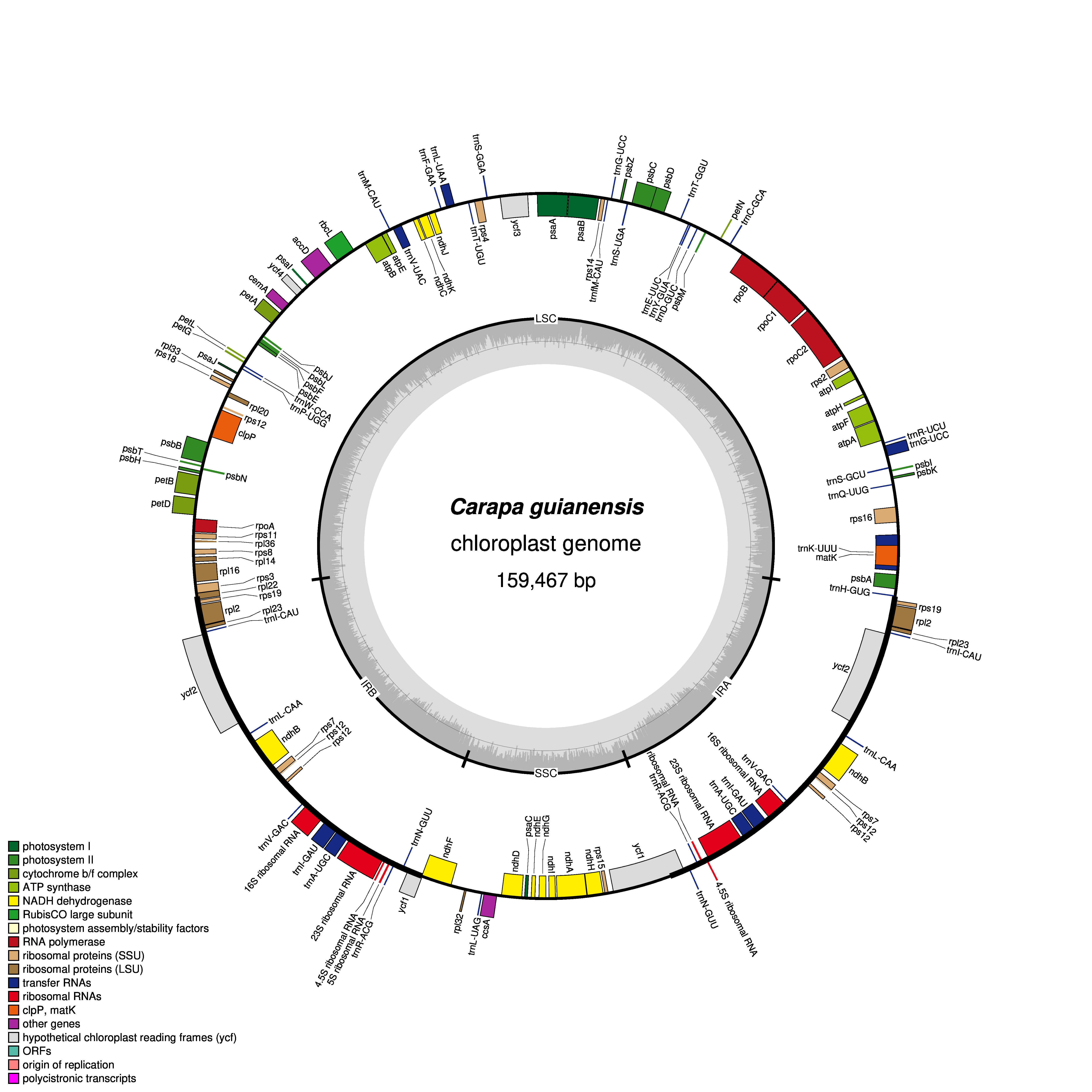

Supplement: Supplemental Information 13 [file peerj-11-15527-s013.jpg]

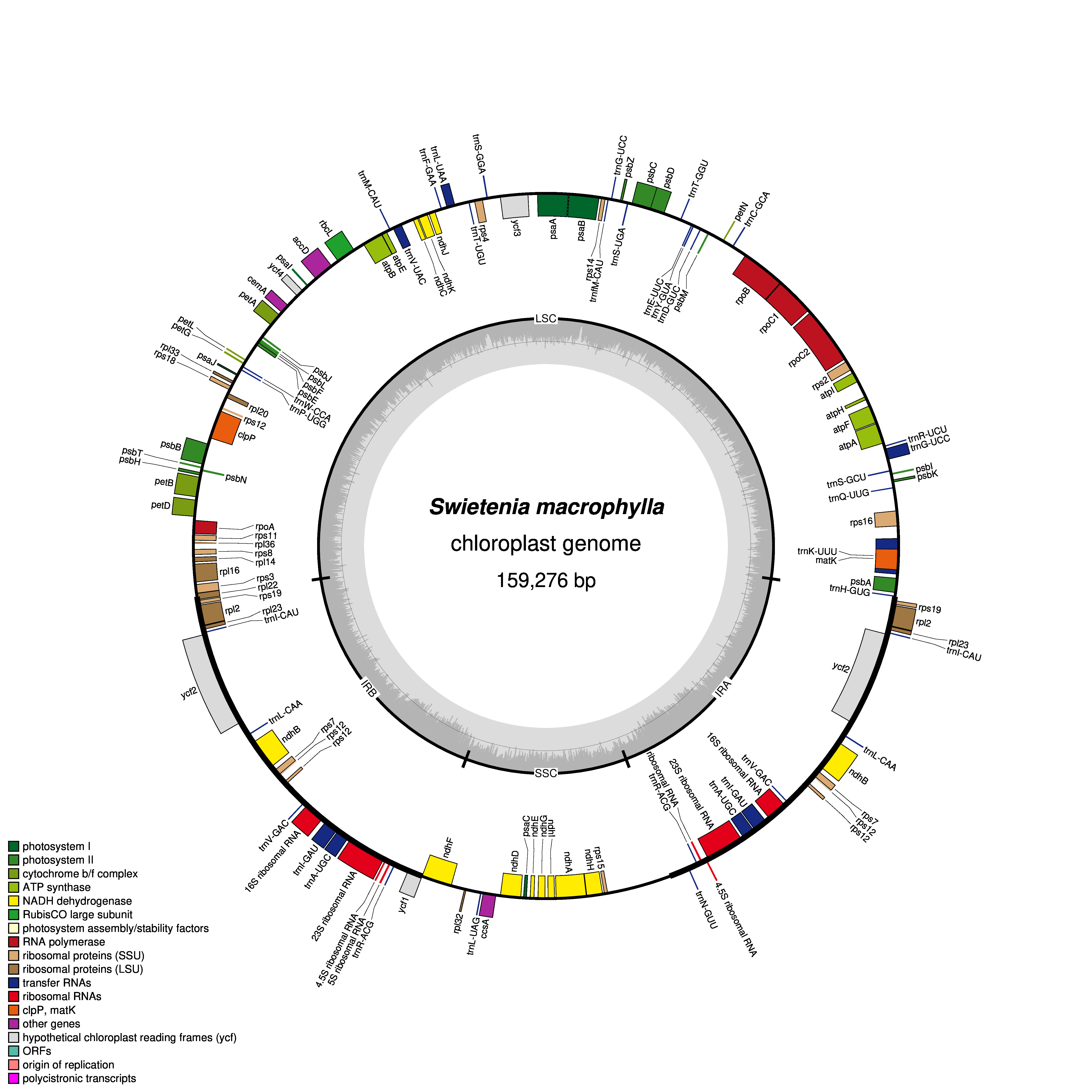

Supplement: Supplemental Information 14 [file peerj-11-15527-s014.jpg]

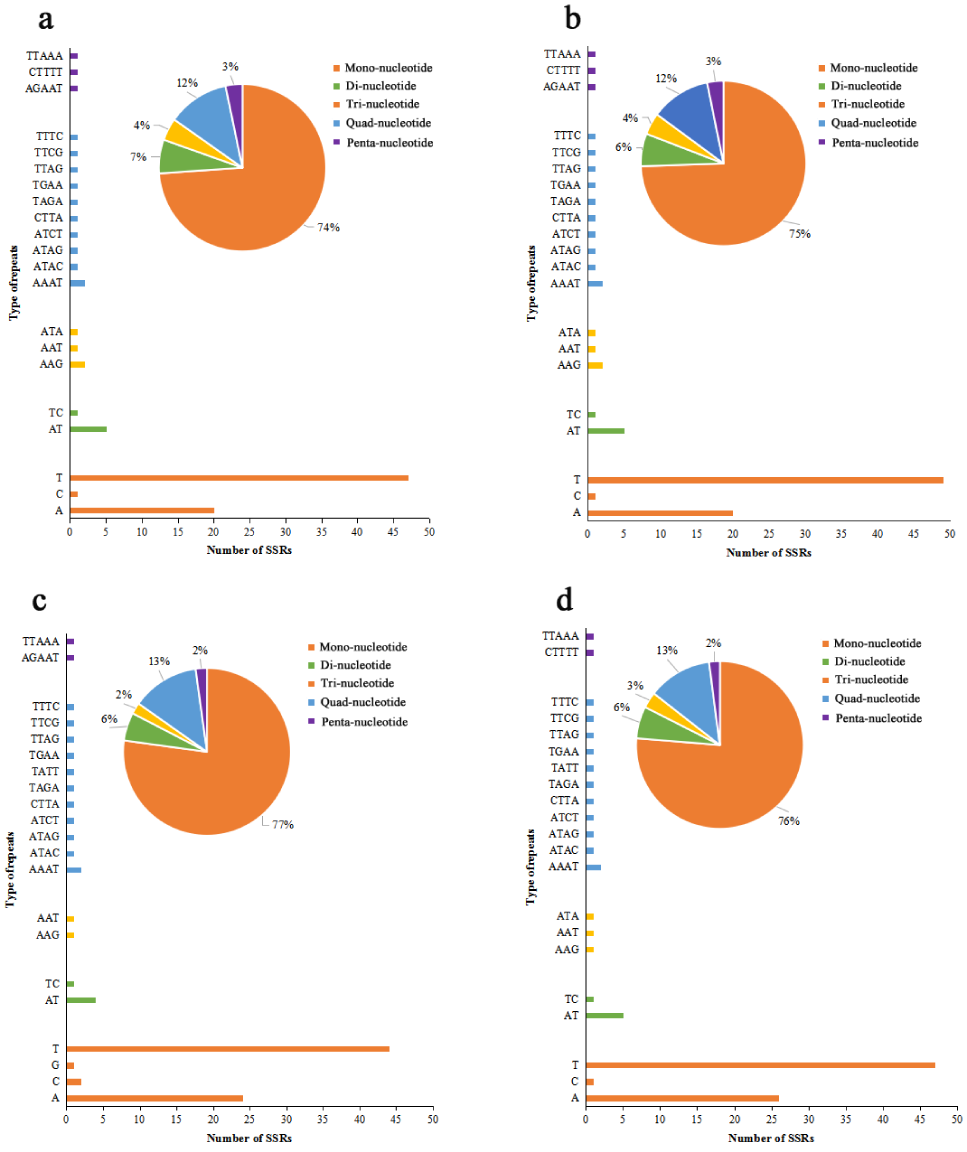

Supplement: Supplemental Information 15 [file peerj-11-15527-s015.tif]
